# Does resource insecurity drive HIV‐related stigma? Associations between food and housing insecurity with HIV‐related stigma in cohort of women living with HIV in Canada

**DOI:** 10.1002/jia2.25913

**Published:** 2022-07-12

**Authors:** Carmen H. Logie, Nina Sokolovic, Mina Kazemi, Shaz Islam, Peggy Frank, Rebecca Gormley, Angela Kaida, Alexandra de Pokomandy, Mona Loutfy, the CHIWOS Research Team

**Affiliations:** ^1^ Factor‐Inwentash Faculty of Social Work University of Toronto Toronto Ontario Canada; ^2^ Women's College Research Institute Women's College Hospital Toronto Ontario Canada; ^3^ Centre for Gender and Sexual Health Equity Vancouver Canada; ^4^ Ontario Institute for Studies in Education University of Toronto Toronto Ontario Canada; ^5^ Alliance for South Asian AIDS Prevention Toronto Ontario Canada; ^6^ Faculty of Health Sciences Simon Fraser University Burnaby British Columbia Canada; ^7^ BC Centre for Excellence in HIV/AIDS Vancouver British Columbia Canada; ^8^ Department of Family Medicine McGill University Montreal Quebec Canada; ^9^ Chronic Viral Illness Service McGill University Health Centre Montreal Quebec Canada; ^10^ Faculty of Medicine University of Toronto Toronto Ontario Canada

**Keywords:** food insecurity, housing insecurity, poverty, stigma, HIV stigma, women living with HIV

## Abstract

**Introduction:**

Women living with HIV across global contexts are disproportionately impacted by food insecurity and housing insecurity. Food and housing insecurity are resource insecurities associated with poorer health outcomes among people living with HIV. Poverty, a deeply stigmatized phenomenon, is a contributing factor towards food and housing insecurity. HIV‐related stigma—the devaluation, mistreatment and constrained access to power and opportunities experienced by people living with HIV—intersects with structural inequities. Few studies, however, have examined food and housing insecurity as drivers of HIV‐related stigma. This study aimed to estimate the associations between food and housing insecurity with HIV‐related stigma among women living with HIV in Canada.

**Methods:**

This prospective cohort study of women living with HIV (≥16 years old) in three provinces in Canada involved three waves of surveys collected at 18‐month intervals between 2013 and 2018. To understand associations between food and housing security and HIV‐related stigma, we conducted linear mixed effects regression models. We adjusted for socio‐demographic characteristics associated with HIV‐related stigma.

**Results and discussion:**

Among participants (*n* = 1422), more than one‐third (*n* = 509; 36%) reported baseline food insecurity and approximately one‐tenth (*n* = 152, 11%) housing insecurity. Mean HIV‐related stigma scores were consistent across waves 1 (mean [*M]* = 57.2, standard deviation [*SD]* = 20.0, *N* = 1401) and 2 (*M* = 57.4, *SD* = 19.0, *N* = 1227) but lower at wave 3 (*M* = 52.8, *SD* = 18.7, *N* = 918). On average, across time, food insecure participants reported HIV‐related stigma scores that were 8.6 points higher (95% confidence interval [CI]: 6.4, 10.8) compared with food secure individuals. Similarly, participants reporting insecure housing at wave 1 tended to experience greater HIV‐related stigma (6.2 points, 95% CI: 2.7, 9.6) over time compared to stably housed participants. There was an interaction between time and housing insecurity, whereby baseline housing insecurity was no longer associated with higher HIV‐related stigma at the third wave.

**Conclusions:**

Among women living with HIV in Canada, experiencing food and housing insecurity was associated with consistently higher levels of HIV‐related stigma. In addition to the urgent need to tackle food and housing insecurity among people living with HIV to optimize wellbeing, getting to the heart of HIV‐related stigma requires identifying and dismantling resource insecurity‐related stigma drivers.

## 1 INTRODUCTION

A robust global evidence base reveals that food and housing insecurity disproportionately impact people living with HIV (PLHIV) [1, 2, 3, 4, 5, 6]. Among PLHIV, food and housing insecurity are linked to poorer HIV outcomes, including unsuppressed viral load, lower CD4 count and poorer physical health [3, 6, 7, 8, 9, 10]. In Canada, out‐of‐pocket costs for publicly funded health services, including antiretroviral therapy (ART), vary by province [11]. This may result in PLHIV needing to apply for social assistance to access comprehensive ART coverage [11, 12]. Limited income from social assistance, low‐wage employment and illness‐related loss of income among employed PLHIV produce challenges acquiring and maintaining sufficient, reliable food and housing [3, 11, 12, 13, 14]. This is not unique to Canada—socio‐economic challenges, including food and housing insecurity, low income and unemployment, were also reported in a review of PLHIV in other high‐income contexts, such as the United States, Australia and the United Kingdom [10]. In addition to being associated with poorer health outcomes [10], food and housing insecurity are resource insecurities linked with poverty, itself a deeply stigmatized phenomenon [15, 16].

Meta‐analytic findings reveal associations between low income and higher HIV‐related stigma [17], signalling the salience of exploring resource insecurity as a driver of HIV‐related stigma. Few quantitative studies have explored this nexus of resource insecurity and HIV‐related stigma [1, 2, 3, 4, 5, 6, 10]. HIV‐related stigma—the devaluation, mistreatment and constrained access to power among PLHIV—intersects with other socially marginalized identities [18, 19, 20], including low socio‐economic status [21]. Among PLHIV experiencing homelessness and housing insecurity in the United States, being recently homeless was associated with increased internalized and perceived HIV‐related stigma [22] and acquiring temporary housing was associated with reduced HIV‐related stigma [23]. A cross‐sectional study in Canada found that concurrent food and housing insecurity was associated with increased HIV‐related stigma among women living with HIV (WLHIV) [24]. Food insecurity was also associated with increased internalized HIV‐related stigma among WLHIV in the United States [7] and higher HIV‐related stigma among PLHIV in Uganda [25, 26]. Together, these studies signal the need to better understand associations between food and housing insecurity with HIV‐related stigma. This understanding could inform downstream interventions to mitigate experiences of intersecting stigma [18, 19], and upstream interventions to transform healthcare and social service environments to reduce stigma exposure [27, 28].

To address knowledge gaps regarding food and housing insecurity as drivers of HIV‐related stigma, this study aimed to estimate the associations between food insecurity and housing insecurity with HIV‐related stigma among a cohort of WLHIV in Canada.

## 2 METHODS

### 2.1 Study setting and population

Data for these analyses came from the Canadian HIV Women's Sexual & Reproductive Health Cohort Study (CHIWOS); methods are described elsewhere [29, 30]. This three‐wave study was conducted in three Canadian provinces (Ontario, Quebec and British Columbia) between 2013 and 2018. Participants were 16 years or older who self‐identified as women and HIV positive and agreed to complete a 2‐hour interviewer‐administered survey three times at 18‐month intervals.

### 2.2 Data collection

At each wave, participants completed a validated 10‐item HIV Stigma Scale [31], which assesses personalized stigma, disclosure concerns, negative self‐image and concern with public attitudes. Total scores were calculated as the sum of scores on each of the 10 items (rated on a 0 to 4 scale) multiplied by 2.5 such that total scores ranged from 0 to 100. The internal consistency of the scale in this study was 0.85.

At baseline, we assessed housing security by asking participants to describe their current place of residence. Individuals living in houses, apartments, self‐contained rooms with amenities or group homes were considered to have secure housing. Individuals living in self‐contained rooms without amenities, transition, halfway or safe houses, in their cars, couch‐surfing or outdoors were considered to have insecure housing. Participants also answered three food security items from the Canadian Community Health Survey Household Food Security Survey (e.g. “in the past 12 months, you and other household members worried that food would run out before you got money to buy more”) [32]. Response options were often ( = 2), sometimes ( = 1) or never true ( = 0), and scores across the three items were summed and dichotomized, wherein a total score of 2 or greater indicated food insecurity. Surveys also included socio‐demographic questions about age, gender identity, sexual orientation, educational attainment and racial/ethnic identity.

### 2.3 Statistical analyses

First, we conducted bivariate tests (Pearson correlations, independent *t*‐tests and two‐way ANOVAs) to examine associations between HIV‐related stigma, food and housing security, and socio‐demographic and health characteristics. Then, we built mixed effects linear regression models with timepoints (level 1) clustered within individuals (level 2). In addition to having random intercepts for individuals, we also included a random coefficient for time to allow trajectories to vary between participants, using an unstructured covariance matrix to avoid imposing constraints on the residual covariances. To understand associations between resource insecurity and stigma over time, we included study wave, food or housing insecurity, and the interaction between wave and resource insecurity as the primary predictors in our models. To control for potential confounding variables, we also included socio‐demographic characteristics that were significantly associated with HIV‐related stigma in bivariate analyses. Missing data were handled using multiple imputation with all participants. All analyses were conducted in Stata v15 (College Station, TX).

### 2.4 Ethical considerations

Informed consent was obtained from participants prior to each survey. Research ethics board (REB) approval was provided by Women's College Hospital, University of Toronto, Simon Fraser University and the University of British Columbia/Providence Health, and McGill University Health Centre. Study sites with independent REBs also obtained their own approval prior to commencing enrolment.

## 3 RESULTS AND DISCUSSION

Sample characteristics at baseline are reported in Table 1. Among participants (*n* = 1422), at baseline more than one‐third (*n* = 509; 36%) reported food insecurity and approximately one‐tenth (*n* = 152, 11%) housing insecurity. Average HIV‐related stigma scores were consistent across waves 1 (*M* = 57.2, *SD* = 20.0, *N* = 1401) and 2 (*M* = 57.4, *SD* = 19.0, *N* = 1227) but lower at wave 3 (*M* = 52.8, *SD* = 18.7, *N* = 918). Participants experiencing food insecurity at baseline were more likely to report significantly higher levels of HIV‐related stigma at all three waves; unstable housing was also significantly associated with greater HIV‐related stigma at the first two waves, but not third (Table 1).

**Table 1:** Sample demographics among a cohort of women living with HIV in Canada (*N* = 1422)

		Association with HIV stigma Pearson *r, t‐* or *F*‐statistic (*p*‐value)		
	Mean (SD) or *N* (%)	Wave 1	Wave 2	Wave 3
Age at baseline	42.8 (10.6)	−0.12 (<0.001)	−0.13 (<0.001)	−0.13 (<0.001)
Months since HIV diagnosis	139.9 (84.6)	−0.13 (<0.001)	−0.18 (<0.001)	−0.10 (0.002)
Gender identity				
Transgender	54 (4%)	0.74 (0.46)	1.22 (0.22)	−0.11 (0.91)
Cisgender	1359 (96%)			
Sexual orientation				
LGBQ2S	180 (13%)	2.23 (0.03)	1.71 (0.09)	2.00 (0.05)
Heterosexual	1237 (87%)			
Educational attainment				
Less than high school	227 (16%)	0.08 (0.94)	−1.35 (0.18)	0.86 (0.39)
High school or greater	1188 (84%)			
Race/ethnicity				
White	584 (41%)	7.28 (<0.001)	11.23 (<0.001)	5.68 (<0.001)
African, Caribbean and Black	418 (29%)			
Indigenous	318 (22%)			
Other or mixed ethnicity	102 (8%)			
Food security				
Food insecure	509 (36%)	8.59 (<0.001)	8.45 (<0.001)	6.54 (<0.001)
Food secure	907 (64%)			
Housing stability				
Unstable housing	152 (11%)	4.23 (<0.001)	2.88 (<0.01)	0.83 (0.41)
Stable housing	1270 (89%)			

Abbreviations: LGBQ2S, lesbian, gay, bisexual, queer or Two‐Spirit; SD, standard deviation.

Individuals who were younger, living with HIV for a shorter time period, identified as lesbian, gay, bisexual, queer or Two‐Spirit (LGBQ2S) and from a racialized group (i.e. Black or Indigenous) also tended to experience greater HIV‐related stigma across waves (Table 1). For example, at baseline, the mean HIV‐related stigma score was 60.3 (*SD* = 19.5) for those who identified as LGBQ2S compared to 56.7 (*SD* = 20.0) for heterosexual participants, and was 60.7 (*SD* = 21.0) for participants who identified as Indigenous and 57.1 (*SD* = 19.0) for participants who identified as African, Caribbean or Black, compared to 54.8 (*SD* = 20.1) for participants identifying as white. As a result, age, months since HIV diagnosis, sexual orientation and race/ethnicity were included as covariates in multivariable analyses.

**Figure 1:**
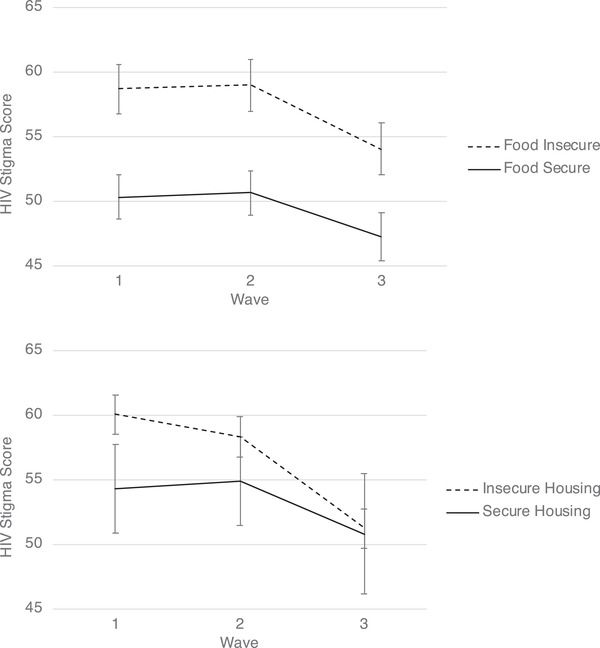
Trajectories of HIV stigma by food insecurity and housing security among a cohort of women living with HIV in Canada. Note: Estimates are for average‐aged, heterosexual, white participants with an average time since HIV diagnosis. Error bars represent 95% confidence intervals.

Results of multilevel regression models are reported in Table 2. Figure 1 presents adjusted scores by time and food insecurity and housing insecurity, along with 95% confidence intervals. At baseline, food insecure individuals reported HIV‐related stigma scores that were 8.35 points higher (95% CI: 6.19, 10.51) than food secure individuals. Similarly, housing insecure participants at wave 1 tended to experience greater HIV‐related stigma (5.72 points, 95% CI: 2.30, 9.15) than those with secure housing. In both models, there was a significant effect of time, whereby stigma scores were significantly lower at wave 3. While those experiencing food insecurity at baseline consistently experienced higher stigma across all three waves, there was a significant interaction between time and housing insecurity, whereby housing insecurity at baseline was no longer associated with higher stigma by the third study wave.

**Table 2:** Longitudinal associations between HIV stigma and food/housing security among a cohort of women living with HIV in Canada (*N* = 1422)

	Food insecurity			Housing insecurity		
	Est.	95% CI	*p*‐value	Est.	95% CI	*p*‐value
Insecure	8.35	6.19, 10.51	<0.001	5.73	2.30, 9.15	0.001
Wave 2	0.34	−1.55, 2.22	0.72	0.56	−0.62, 1.73	0.35
Wave 3	−3.09	−5.19, −1.00	0.004	−3.51	−4.80, −2.21	<0.001
Insecure × Wave 2	−0.05	−2.36, 2.26	0.97	−2.31	−5.93, 1.32	0.21
Insecure × Wave 3	−1.54	−4.22, 1.13	0.26	−5.31	−10.37, −0.26	0.04

Note: Models adjusted for age, months since HIV diagnosis, sexual orientation and race/ethnicity.

In this study with WLHIV in Canada, we found that experiencing food or housing insecurity was associated with significantly higher levels of HIV‐related stigma. This suggests that resource insecurity‐related factors increase exposure to HIV‐related stigma. These findings corroborate research on associations between HIV‐related stigma and food insecurity in both high‐income [2, 7, 33] and low‐ and middle‐income contexts [25, 26], and between HIV‐related stigma and housing insecurity in high‐income contexts [22]. Despite calls to harness poverty alleviation to reduce HIV‐related stigma in sub‐Saharan Africa nearly a decade ago [34], there is limited research on poverty and resource scarcities as stigma drivers in sub‐Saharan Africa, particularly with WLHIV who are most impacted by the pandemic.

Poverty contributes to food and housing insecurity [35, 36], and our findings build on the evidence base that poverty is associated with increased HIV‐related stigma [7, 17, 22, 24]. Stigma towards poverty is rooted in social constructions that it symbolizes “failure,” “laziness” and “irresponsibility” [15, 37, 38]. Homelessness may be stigmatized more than poverty due to its visibility and perceived disruptiveness of public space [39]. Food insecurity can result in eating stigmatized foods and acquiring food through socially unacceptable means, resulting in blame, shame and social isolation [40, 41]. Our findings highlight the utility of applying a resource insecurity framework [41] to HIV‐related stigma research.

Poverty is relational, involving social and institutional mistreatment, as well a core experience that involves disempowerment, suffering and struggle [42, 43, 44]. The “pathologization of poverty” (p. 78) [15], the ways in which people receiving disability‐related benefits are stigmatized, is rooted in local moral economies where unemployment is socially devalued [15, 16, 45]. For instance, PLHIV who receive food assistance may experience social assistance services as uncompassionate and penalizing, while simultaneously feeling judged by society for being ill *and* for receiving social assistance [16]. It is plausible that these experiences heighten HIV‐related stigma, itself rooted in moral judgement [46]. These findings on resource insecurity, alongside findings that lesbian, gay, bisexual and queer (LGBQ) and racialized participants experienced higher HIV‐related stigma, reflect an intersectional experience of stigma [18, 19, 20], whereby social categories coalesce at the individual level of experience and expose interlocking systems of oppression [47].

HIV‐related stigma reduced among participants over time, and stigma disparities by housing status also attenuated over time. Earnshaw et al. describe stigma as fluid and dynamic, situating stigma changes within historical context, developmental period and stigmatized status course [48]. For instance, our finding that younger participants reported higher HIV‐related stigma could be understood from a developmental perspective, whereby younger persons undergo social transitions in education, employment and relationships that could present different stigma exposures [48]. Time since diagnosis was associated with reduced HIV‐related stigma, aligning with the status course timescale, whereby persons can acquire stigma resilience, coping, self‐esteem, self‐efficacy and social support over time [48]. It is plausible that housing insecure participants at baseline were housed over time, as prior research documented that help‐seeking self‐efficacy and time living with HIV were associated with attaining stable housing over time [49]. Increased social support over time can also result in stigma reduction and assistance meeting housing needs [50].

### 3.1 Strengths and limitations

There are three main limitations. First, we only collected housing and food insecurity data at baseline, and did not ask about duration of housing/food insecurity status. Thus, findings only tell us about how these indicators, assessed at one timepoint, affect later outcomes. Second, stigma is intersectional [19, 20, 21, 51], and we only examined HIV‐related stigma. Third, we did not assess why HIV‐related stigma reduced over time; historical contexts include social change movements, such as undetectable = untransmittable, that may reduce HIV‐related stigma [48, 52, 53].

## 4 CONCLUSIONS

Tackling resource insecurity is necessary to get to the heart of HIV‐related stigma. HIV‐related stigma and its relationships with resource insecurity require multi‐faceted approaches. Structural interventions can address PLHIV's employment barriers, including workplace stigma, universal ART coverage and unpredictable periodic disability [54, 55]. Addressing housing insecurity could be integral to reducing HIV‐related stigma [23]. As food and housing insecurity are associated with social isolation [3, 4, 56], strategies can leverage community solidarity and support [57]. Holistic approach to care can address poverty‐related challenges [44], such as offering comprehensive and medically appropriate food support [58]. Advancing structural competency [59] and strengths‐focused, client‐centred clinical care [23] may produce social change for WLHIV.

## COMPETING INTERESTS

None to declare.

## AUTHORS’ CONTRIBUTIONS

CHL conceptualized paper and led writing. NS conducted analyses and contributed to writing. MK, SI, PK, ML, AK, AdP and ML provided edits and contributed to study design and implementation. All other CHIWOS Research Team^ members contributed to study design and supported data collection.

## FUNDING

CHIWOS Research Team: Rahma Abdul‐Noor (Women's College Research Institute), Aranka Anema (Harvard Medical School), Jonathan Angel (Ottawa Hospital Research Institute), Dada Mamvula Bakombo (McGill University Health Centre), Fatimatou Barry (Women's College Research Institute), Greta Bauer (University of Western Ontario), Kerrigan Beaver (Women's College Research Institute), Marc Boucher (CHU Ste‐Justine), Isabelle Boucoiran (CHU Ste‐Justine), Jason Brophy (Children's Hospital of Eastern Ontario), Lori Brotto (University of British Columbia), Ann Burchell (St, Michael's Hospital), Claudette Cardinal (Simon Fraser University), Allison Carter (Kirby Institute), Lynne Cioppa (Women's College Research Institute), Tracey Conway (Women's College Research Institute), José Côté (Centre Hospitalier de l'Université de Montréal), Jasmine Cotnam (Canadian Aboriginal AIDS Network), Cori d'Ambrumenil (AIDS Vancouver Island), Janice Dayle, (McGill University Health Centre), Erin Ding (British Columbia Centre for Excellence in HIV/AIDS), Danièle Dubuc, (McGill University Health Centre), Janice Duddy (Pacific AIDS Network), Mylène Fernet (Université du Québec à Montréal), Annette Fraleigh (Women's College Research Institute), Peggy Frank (Simon Fraser University), Brenda Gagnier (Women's College Research Institute), Marilou Gagnon (University of Victoria), Jacqueline Gahagan (Dalhousie University), Claudine Gasingirwa (Women's College Research Institute), Nada Gataric (British Columbia Centre for Excellence in HIV/AIDS), Rebecca Gormley (British Columbia Centre for Excellence in HIV/AIDS), Saara Greene (McMaster University), Danielle Groleau (McGill University), Charlotte Guerlotté (COCQ‐SIDA), Trevor Hart (Ryerson University), Catherine Hankins (McGill University), Roula Hawa (Women's College Research Institute), Emily Heer (Alberta Health Services), Robert S. Hogg (Simon Fraser University), Terry Howard (Glasshouse Consultants), Joseph Jean‐Gilles (GAP‐VIES), Hermione Jefferis (AIDS Vancouver Island), Evin Jones (Pacific AIDS Network), Charu Kaushic (McMaster University), Mary Kestler (Oak Tree Clinic BCWH) Maxime Kiboyogo (McGill University Health Centre), Marina Klein (McGill University Health Centre), Nadine Kronfli (McGill University Health Center), Gladys Kwaramba (Women's College Research Institute), Gary Lacasse (Canadian AIDS Society), Ashley Lacombe‐Duncan (University of Michigan), Melanie Lee (Simon Fraser University), Rebecca Lee (CIHR Canadian HIV Trials Network), Jenny Li (British Columbia Centre for Excellence in HIV/AIDS), Viviane Lima (British Columbia Centre for Excellence in HIV/AIDS), Elisa Lloyd‐Smith (Vancouver General Hospital), Carmen Logie (University of Toronto), Evelyn Maan (Oak Tree Clinic), Valérie Martel‐Lafrenière (Centre Hospitalier de l'Université de Montréal), Carrie Martin (Canadian Aboriginal AIDS Network), Renee Masching (Canadian Aboriginal AIDS Network), Lyne Massie (Université du Québec à Montréal), Melissa Medjuck (formerly of the Positive Women's Network), Brigitte Ménard, (McGill University Health Centre), Cari L. Miller (formerly of Simon Fraser University), Judy Mitchell (Positive Living North), Gerardo Mondragon (British Columbia Centre for Excellence), Deborah Money (Faculty of Medicine at UBC), Ken Monteith (COCQ‐SIDA), Marvelous Muchenje (Women's Health in Women's Hands CHC), Florida Mukandamutsa (CASM), Mary Ndung'u (African Partnership Against AIDS), Valerie Nicholson (Simon Fraser University), Kelly O'Brien (University of Toronto), Nadia O'Brien (McGill University Health Centre and McGill University), Gina Ogilvie (University of British Columbia), Susanna Ogunnaike‐Cooke (Public Health Agency of Canada), Joanne Otis (Université du Québec à Montréal), Rebeccah Parry (Simon Fraser University), Sophie Patterson (Simon Fraser University), Angela Paul (Positive Living North), Doris Peltier (Canadian Aboriginal AIDS Network), Neora Pick (Oak Tree Clinic BCWH), Alie Pierre (McGill University Health Centre), Jeff Powis (Michael Garron Hospital), Karène Proulx‐Boucher (McGill University Health Centre), Corinna Quan (Windsor Regional Hospital), Jesleen Rana (Women's Health in Women's Hands CHC), Eric Roth (University of Victoria), Danielle Rouleau (Centre Hospitalier de l'Université de Montréal), Geneviève Rouleau (Centre Hospitalier de l'Université de Montréal), Sergio Rueda (Centre for Addiction and Metal Health), Kate Salters (British Columbia Centre for Excellence in HIV/AIDS), Margarite Sanchez (ViVA), Roger Sandre (Haven Clinic), Jacquie Sas (CIHR Canadian HIV Trials Network), Édénia Savoie (McGill University Health Centre), Paul Sereda (British Columbia Centre for Excellence in HIV/AIDS), Stephanie Smith (Women's College Research Institute), Marcie Summers (formerly of the Positive Women's Network), Wangari Tharao (Women's Health in Women's Hands CHC), Christina Tom (Simon Fraser University), Cécile Tremblay (Centre Hospitalier de l'Université de Montréal), Jason Trigg (British Columbia Centre for Excellence in HIV/AIDS), Sylvie Trottier (Centre Hospitalier Universitaire de Québec), Angela Underhill (Women's College Research Institute), Anne Wagner (Ryerson University), Sharon Walmsley (University Health Network), Clara Wang (British Columbia Centre for Excellence in HIV/AIDS), Kath Webster (Simon Fraser University), Wendy Wobeser (Queen's University), Denise Wozniak (Positive Living Society of British Columbia), Mark Yudin (St. Michael's Hospital), Wendy Zhang (British Columbia Centre for Excellence in HIV/AIDS), Julia Zhu (British Columbia Centre for Excellence in HIV/AIDS). All other CHIWOS Research Team Members who wish to remain anonymous.

## Data Availability

Data are available from the Women’s College Research Institute Women and HIV Research Program Data Access Coordinator for researchers and students who meet the research ethics board criteria for accessing confidential data. The current Data Access Coordinator is Jill Koebel and she can be reached at jill.koebel@wchospital.ca. The criteria for access to the confidential data include (1) being added as a CHIWOS researcher or student to the research ethics board (REB) application and (2) signing a CHIWOS Data Sharing and Collaboration Agreement. The de‐identified data set cannot be publicly shared at this point as we do not have community or REB approval to do so. Co‐authorship is a requirement for data access as per the CHIWOS authorship policy, which includes the requirement that the ICMJE authorship criteria be met by all authors.
